# Novel electrodiagnostic provocative techniques for the diagnosis of suspected tarsal tunnel syndrome

**DOI:** 10.1186/s43166-022-00151-z

**Published:** 2022-09-30

**Authors:** Mona M. Hasab ElNaby, Amr Farouk Abdel Rahman, Rehab Ali Ibrahim

**Affiliations:** 1 Physical medicine, Rheumatology and Rehabilitation Department, Faculty of Medicine, Ain Shams University, Cairo, Egypt; 2 Orthopedic Surgery and Traumatology Department, Ain Shams University Hospitals, Cairo, Egypt

**Keywords:** Foot pain, Tarsal tunnel syndrome, Standing, NCSs, Provocative, Dorsiflexion, Eversion, Medial plantar, Lateral plantar

## Abstract

**Background:**

Electrodiagnostic tests  (EDXTs) have been considered the gold standard method for the diagnosis of tarsal tunnel syndrome (TTS); however, definitive tests has not yet been discovered. Our aim was to develop new nerve conduction provocative techniques in the double and single leg stance as well as combined ankle dorsiflexion with foot eversion accompanied by compression for the diagnosis of suspected TTS.

**Results:**

Routine combined nerve conduction studies (NCSs) using medial plantar (MP) and lateral plantar (LP) nerves had a 60.9% sensitivity for the diagnosis of TTS. The diagnostic sensitivity increased when combining the three novel tests reaching 82.6% and 78.3% using either MP or LP NCSs respectively. The diagnostic sensitivity further increased to reach 100% when combining the MP and LP novel NCSs considering either the latency or amplitude.

**Conclusion:**

The full diagnostic sensitivity for TTS reached 100% by using a battery of novel tests. The proposed diagnostic workup by this study recommends using these biomechanically challenging tests to complete the diagnostic battery of tests especially in symptomatic patients with negative routine tests.

## Background

Foot pain is a common disorder reported in 24% of adults and can be a cause of disability [1]. Among the causes of foot pain is the entrapment of tibial nerve or one of its branches a condition known as tarsal tunnel syndrome (TTS) [2]. According to literature, this condition is considered uncommon, and its diagnosis seems to be difficult [3]. Diagnosis of TTS is suspected by the patient’s symptoms and established based on electrodiagnostic tests (EDXTs) that have been considered a gold standard test [4]. Different electrodiagnostic (EDX) approaches tried to confirm a clinically suspected TTS, however definitive tests to confirm TTS has not yet been discovered [5]. Previous studies tried to develop new techniques for stimulation of TTS symptoms during nerve conduction studies (NCSs). In 2012, Abouelela and Zohiery developed the triple compression stress test (TCST), to elicit stress on posterior tibial nerve and its branches, to aggravate signs of its entrapment [6]. The idea of inducing TTS symptoms by compressing the ankle and subtalar joints while straining them seems reasonable, however reviewing the literature revealed that there are other positions that appear to be more biomechanically challenging to the foot. During standing the compression of the distal part of the tibial and plantar nerves increase due to its contact with the medial process of the talus [7]. Also, foot eversion and ankle dorsiflexion can exacerbate the symptoms. Pressure on the medial side of the ankle along the course of the tibial nerve is believed to be painful in 60 to 100% of those affected and to worsen paresthesia [8]. To the best of our knowledge, routine EDXTs for the diagnosis of suspected TTS are usually done in the supine position and this is the first time to conduct NCSs for the MP and LP nerves during standing and on combined foot eversion and ankle dorsiflexion to develop new provocative, sensitive, and objective tests to help in the diagnosis of suspected cases.

Finding an easy, reliable EDX procedure to confirm the diagnosis of suspected TTS remains a challenge. Our aim was to develop new nerve conduction provocative techniques in the double and single leg stance as well as combined ankle dorsiflexion and foot eversion accompanied by compression for the diagnosis of suspected TTS.

## Methods

### Study design, setting, and participants

This was a case control study with two control groups. The study was conducted in the electro-diagnostic unit of the Physical Medicine, Rheumatology and Rehabilitation department at Ain Shams University hospitals. Participants were recruited from orthopedic department (foot and ankle unit) and physical medicine, rheumatology, and rehabilitation outpatient clinics at **** University hospitals.

### Participants

The study included three groups, a patient group (group I) and two control groups (group IIa and group IIb). The patient group included thirty patients with foot pain either unilateral or bilateral fulfilling the inclusion criteria. Two control groups were enrolled in the study each included 15 matched individuals (30 feet) giving a total number of 60 feet. Group IIa included normal healthy individuals and group IIb included patients complaining of foot pain due to other conditions not meeting TTS symptoms or signs.

Inclusion criteria were adult patients with foot pain, tingling, numbness and or dysesthesia suggestive of TTS in one or both feet (history of pain and numbness in sole of foot and ankle exacerbated with standing or walking). Patients with inability to stand or having a history of recent intra-articular corticosteroid injection to the foot or ankle, or those with body mass index (BMI) ≥ 35 were excluded. Patients with neurological disorders causing tingling and numbness of the foot especially peripheral neuropathy and radiculopathy were excluded, external pacing wires and radiculopathy or other causes of pain were also excluded.

### All participants underwent

Recording for age, sex, and BMI. Full medical history was obtained including any history of foot pain, tingling, numbness, and/ or dysesthesia that could be suggestive of TTS and whether symptoms were exacerbated by standing or walking. The duration of illness, smoking history, previous foot injury and any previous attempts to alleviate foot pain with medications, injection, supporting devices, physical therapy, or surgery were also recorded.

Patients were subjected to general and musculoskeletal examination including foot and ankle and neurological assessment.

Tinel’s test was done and recorded (as positive or negative).

### Electrodiagnostic studies

Electrodiagnostic studies were conducted using Nihon Kohden (Japan), MEB-9400, EDX equipment.

### Routine motor nerve conduction studies (MNCSs)

Routine MNCSs for both MP, LP including F wave for tibial nerve and MNCSs for peroneal nerves were done bilaterally for patients and controls. Limb temperature was maintained at 33 °C. Surface electrodes were used for both stimulation and recording. Recording was done from abductor hallucis (AH) and abductor digiti quinti (ADQ) for MP and LP nerves respectively and extensor digitorum brevis (EDB) for the peroneal nerve. The latency and amplitude of motor response were recorded and interpreted according to Preston and Shapiro, 2021 [9]. The sites of stimulating and recording electrodes were marked in the supine position.

The patient was diagnosed as having TTS if one of the following criteria was obtained on doing the routine basic tests according to Preston and Shapiro, 2021 [9]:

- Distal motor latency of MP nerve is > 5.8 ms.
- Distal motor latency of LP nerve is > 6.3 ms.
- Amplitude of compound motor action potential < 4 mv for AH muscle or < 3 mv for the ADQ muscle.
- Side to side amplitude difference more than 50% of the same nerve branch.

Routine sensory nerve conduction studies for sural nerve were done to cases and controls. Those with abnormal peroneal compound motor action potential and/or sural sensory nerve action potential were excluded from the study as this might point out to another diagnosis beyond TTS.

### Novel provocative testing

**Fig. 1:**
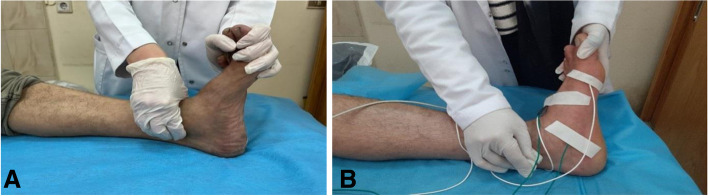
EDCT (**A**) Compression of the tibial artery for 30 s during eversion of the foot and dorsiflexion of the ankle, (**B**) NCSs of the medial plantar (MP) nerve using novel provocative test with eversion of the foot and dorsiflexion of the ankle

**Fig. 2:**
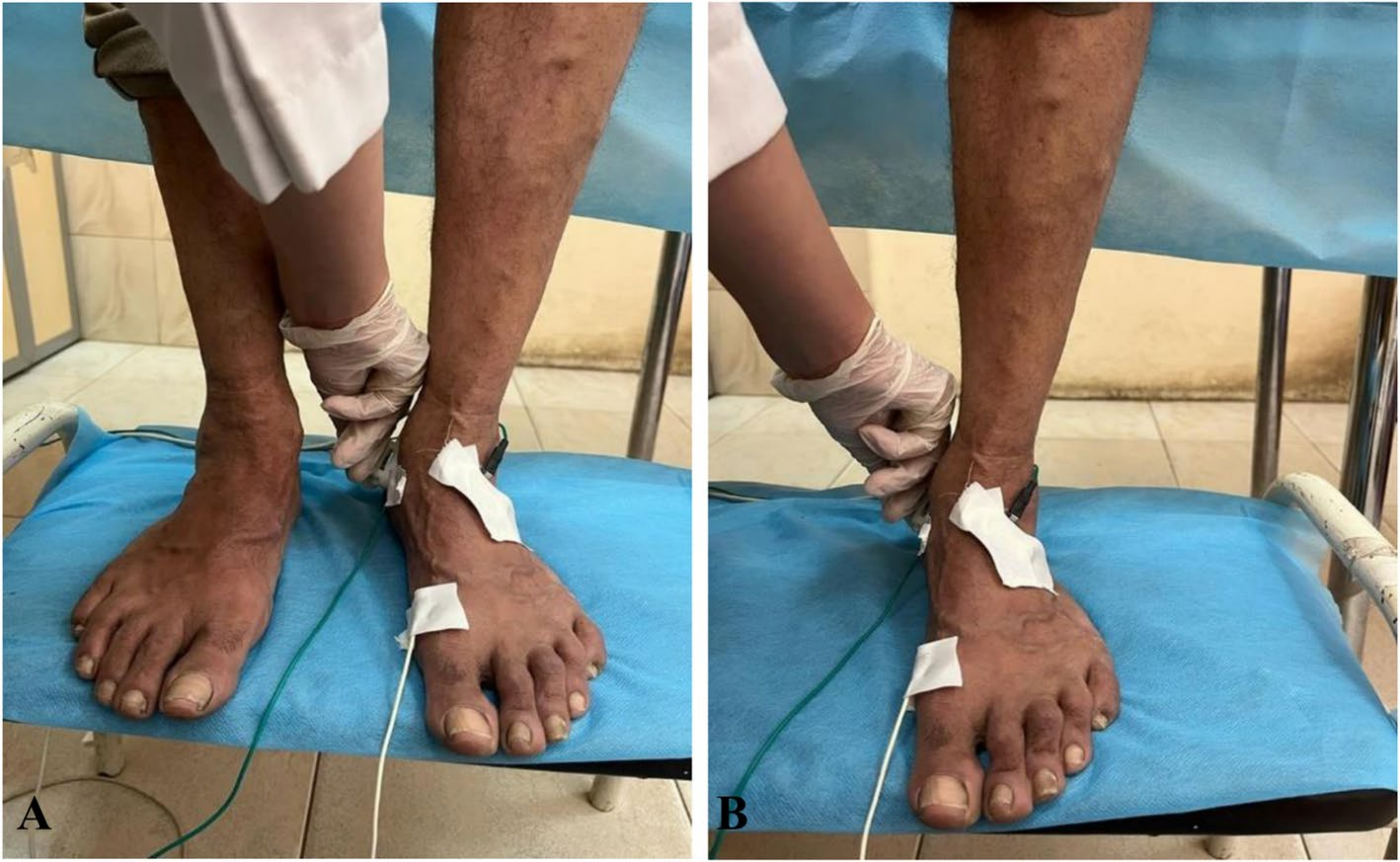
NCSs of Medial plantar (MP) nerve using novel test during (**A**) double stance (DS) using DST, and (**B**) single stance (SS) using SST

MNCSs for MP and LP nerves were done (at the same sites marked during the supine position) with provocation during:

- Supine position using eversion dorsiflexion compression test (EDCT) with eversion of the foot and dorsiflexion of the ankle with added compression for the tibial artery just above the medial malleolus for 30 s (Fig. 1).
- Standing MNCSs for both MP and LP nerves with the same parameters were recorded during standing in double stance (DS) using double stance test (DST), and in single stance (SS) using single stance test (SST) (Fig. 2). The recording was made after acquiring standing for 30 s. The patient received standby support by an assistant if needed to acquire balance on one foot for 30 s then response is obtained.

### Ethics approval and consent to participate

Ethical Approval for the study was obtained from Ain Shams  University, Faculty of Medicine Research Ethics Committee (REC) FWA 000017585. FMASU R 122/2020. A written informed consent was obtained from all participants to contribute in this study.

### Statistical analysis

Statistical analysis was done using SPSS version 25. Quantitative data were presented as mean and standard deviation and two independent samples *t* test was used to test the significance of difference between means. Qualitative data were presented as count and proportion. ROC curve analysis was done and AUC as well as sensitivity, specificity. LR for a positive and negative result have been calculated with their 95% confidence intervals. All point estimates will be accompanied by 95% confidence limits and for hypothesis testing *P* values ≤ 0.05 will be considered statistically significant

## Results

Patients had mean age of 48.8 ± 12.03 years while controls’ mean age was 43.2 ± 12.44 and the difference was statistically insignificant. BMI mean was 27.62 ± 3.96 for cases and 29.46 ± 4.72 for controls and the difference was statistically insignificant. The mean duration of the disease among cases was 16.57 ± 8.79 months. The sex distribution was equal between the patient and both control groups with 50% males in each.

Symptoms as described by our patients (46 feet) were foot pain, tingling and/or numbness. Symptoms were exacerbated by standing or walking in 19 patients (25 feet). Only 37 feet had positive Tinel’s test. Twelve cases (21 feet) showed pronated feet on inspection, 9 of them were bilateral. Group IIb were referred for foot pain, none of them had symptoms of TTS. The main causes for their foot pain were plantar fasciitis in 5 patients, 3 Achilles tendinitis, 2 metatarsalgia, 3 post-fracture metatarsal bone and 2 Hallux valgus.

Routine EDXTs were positive for TTS in only 28 feet with a sensitivity of up to 60.9% when MP and LP NCSs were combined with either drop in amplitude and or an increase in latency. This means that the routine tests were unable to diagnose 18 feet with clinically suspected TTS. The sensitivity of the routine NCSs is shown in (Table 1) for MP and LP nerves separately considering amplitude and latency. NCSs showed 45.7% sensitivity for diagnosis of TTS when one of the two branches (MP and LP nerves) is affected (drop in amplitude and /or delay in latency) (Table 1).

**Table 1:** Number of positive cases and percentage of positive routine NCSs among all cases

	No. (46)	%
MP amplitude	6	13.0
MP latency	17	37.0
LP amplitude	13	28.3
LP latency	15	32.6
MP (amplitude/latency)	21	45.7
LP (amplitude/latency)	21	45.7
Nerve affection (MP + LP)	28	60.9

*NCSs* nerve conduction studies, *No* Number of cases, *MP* Medial plantar, and *LP* Lateral plantar

Table 2 shows the number and the percentage of symptomatic cases who were diagnosed by the novel tests despite showing values within normal on routine testing for (amplitude/latency) MP or LP.

**Table 2:** Number (No.) and percentage (%) of positive results by the novel tests among those with negative routine tests for diagnosis of TTS

	Normal MP amplitude by routine tests (No.:40)			Normal LP amplitude by routine tests (No.:33)	
Amplitude	No.	%	Amplitude	No.	%
MP_EDCT	28	70.0	LP_EDCT	28	84.8
MP_DST	24	60.0	LP_DST	25	75.8
MP_SST	25	62.5	LP_SST	21	63.6
	Normal MP latency by routine tests (No.: 29)			Normal LP latency by routine tests (No.: 31)	
Latency	No.	%	Latency	No.	%
MP_EDCT	22	75.9	LP_EDCT	25	80.6
MP_DST	15	51.7	LP_DST	22	71.0
MP_SST	14	48.3	LP_SST	20	64.5

*TTS* Tarsal tunnel syndrome, *MP* Medial plantar, *LP* Lateral plantar, *EDCT* Eversion dorsiflexion compression test, *DST* Double stance test, *SST* Single stance test

Only 37 feet had positive Tinel’s test while 9 feet had negative Tinel’s test. Eight of them had negative routine test while they all proved positive by the battery of novel tests. This gives the novel tests the advantage of confirming the affection TTS in the absence of provocative clinical Tinel’s test.

**Fig. 3:**
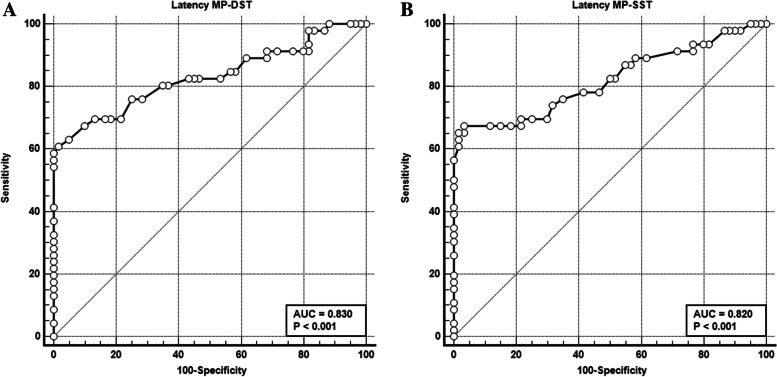
Excellent discrimination of MP latency stance between the healthy and diseased feet during **A** double stance (AUC = 0.830). **B** Single stance (AUC = 0.82)

ROC curve analysis of the novel tests (Table 3, Fig. 3) showed excellent discrimination of MP latency during DS (60.8%) and single stance (76.3%) between the healthy and diseased feet (AUC = 0.83, 0.82 respectively), while LP latency during double and single stance showed acceptable discrimination between cases and controls (AUC 0.74). The cut-off value of MP latency during DS and SS was > 5.9 and > 5.8 respectively.

**Table 3:** Cut-off values, specificity, and sensitivity of the novel tests based on ROC curve analysis comparing cases with controls

	Criterion	Sensitivity	95% CI	Specificity	95% CI	+LR	95% CI	−LR	95% CI
**Amplitude** **MP_EDCT**	≤ 6.2	39.13	25.1–54.6	91.67	81.6–97.2	4.70	1.88–11.70	0.66	0.52–0.85
**Latency MP_EDCT**	> 5.5	52.17	36.9–67.1	96.67	88.5–99.6	15.65	3.90–62.87	0.49	0.36–0.67
**Amplitude** **LP_EDCT**	≤ 2.9	36.96	23.2–52.5	100.00	93.9–100			0.63	0.51–0.79
**Latency LP_EDCT**	> 6.3	45.65	30.9–61.0	100.00	93.9–100			0.54	0.42–0.71
**Amplitude** **MP_DST**	≤ 6.6	47.83	32.9–63.1	80.00	67.7–89.2	2.39	1.33–4.31	0.65	0.48–0.88
**Latency MP_DST**	> 5.9	60.87	45.4–74.9	98.33	91.1–100	36.52	5.16–258.6	0.40	0.28–0.57
**Amplitude** **LP_DST**	≤ 2.9	43.48	28.9–58.9	100.00	94.0–100			0.57	0.44–0.73
**Latency LP_DST**	> 6.3	50.00	34.9–65.1	100.00	94.0–100			0.50	0.37–0.67
**Amplitude** **MP_SST**	< 6	45.65	30.9–61.0	80%	76.7–89.2	2.28	1.26–4.14	0.68	0.51–0.91
**Latency MP_SST**	> 5.8	67.39	52.0–80.5	96.67	88.5–99.6	20.22	5.10–80.15	0.34	0.22–0.51
**Amplitude** **LP_SST**	≤ 2.86	52.17	36.9–67.1	100.00	94.0–100			0.48	0.35–0.65
**Latency LP_SST**	> 6.3	54.35	39.0–69.1	100.00	94.0–100.			0.46	0.33–0.63

*MP* Medial plantar, *LP* Lateral plantar, *DST* Double stance test, *SST* Single stance test, *EDCT* Eversion dorsiflexion compression test

**Fig. 4:**
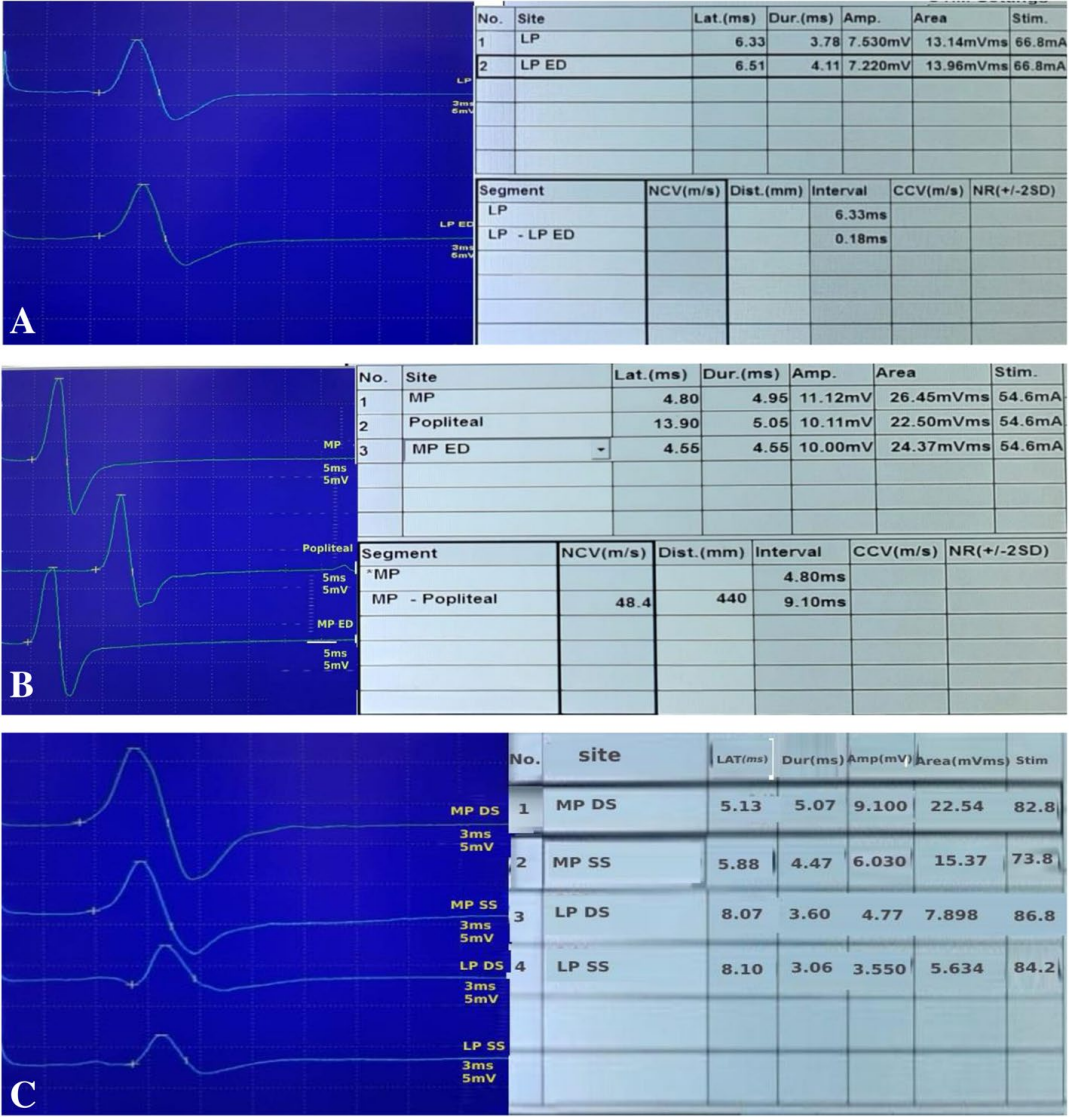
The figure shows the novel tests can diagnose TTS in a symptomatic patient who has pronated flat feet with normal motor latency and amplitude of left (**A**) lateral plantar (LP) and, (**B**) medial plantar (MP) nerves by routine NCSs which showed an increase in latency and decrease in amplitude by the novel tests (ED, SS, and DS) more marked with the last 2 tests (**A**, **C**)

**Fig. 5:**
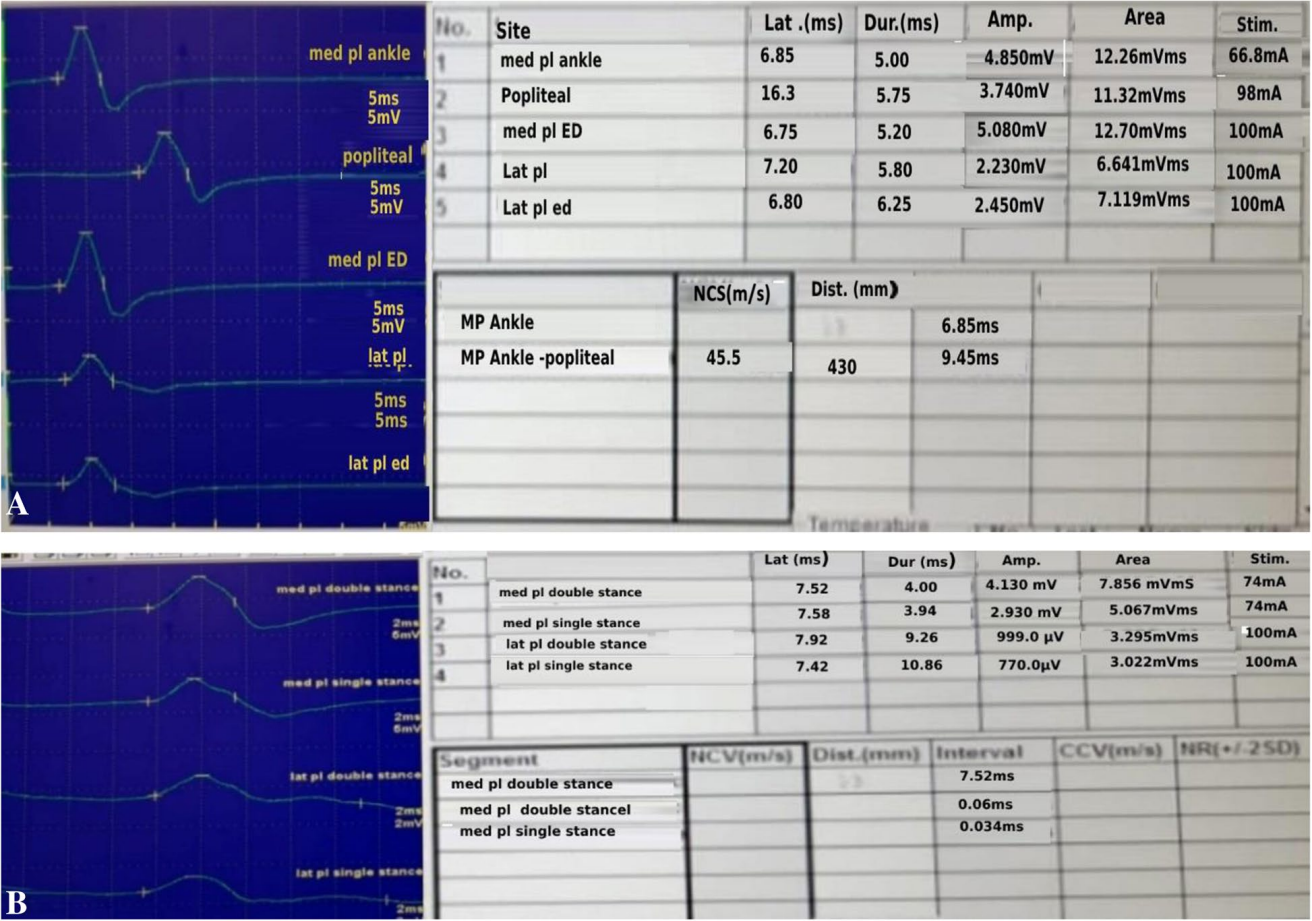
NCSs of MP and LP nerves in a 20-year-old adult with tarsal coalition using routine NCSs and Novel tests (EDCT, DST, SST) showing delayed latency of MP and LP nerves with decreased amplitude of LP nerve using routine tests (**A**), which was confirmed by novel tests specially MP SST where not only more latency delay was noticed but also marked drop of the amplitude of M response (**B**). These findings highlight how the novel tests could confirm the diagnosis of TTS by exaggerating the mild affection seen on doing routine NCSs by biomechanically challenging the nerve using stance positions (DST and SST)

On the other hand, MP and LP amplitude showed poor discrimination at double and single stance (AUC = 0.62, 0.64 for MP& 0.63, 0.67 for the LP respectively). Also, regarding MP amplitude and latency in ED and LP latency there was poor discrimination (AUC 0.61, 0.59, 0.664 respectively).

As specificity of the new tests was high, we tried to test the sensitivity based on combination of the tests. The sensitivity of combination of NCSs of both MP and LP nerves using the novel tests (EDCT, DST, SST) showed 100% sensitivity and was able to diagnose all the 46 symptomatic feet followed by NCSs of MP with affection of either amplitude or latency which showed 82.6% sensitivity and was able to detect TTS in 38 feet. LP NCSs using novel tests was able to detect TTS in 36 feet with 78.3 % sensitivity if the amplitude or latency were affected in one of the three novel tests (Table 4, Figs. 4 and 5).

**Table 4:** The sensitivity of the novel tests when combined to increase the diagnostic sensitivity

Parameter/s of the novel tests (EDCT/DST/SST)	No. of positive cases	%
Amplitude MP	25	54.3%
Amplitude LP	26	56.5%
Latency MP	32	69.6%
Latency LP	26	56.5%
MP (amplitude or latency)	38	82.6%
LP (amplitude or latency)	36	78.3%
MP or LP	46	100.0%

*EDCT* Eversion dorsiflexion compression test, *DST* Double stance test, *SST* Single stance test, *MP* Medial plantar, *LP* Lateral plantar

## Discussion

The diagnosis of TTS is suspected on clinical examination and confirmed by EDX evaluation; however, routine NCSs cannot diagnose a considerable number of cases with a percentage of false-negative results creating a challenge for the treating physician. Despite efforts to develop diagnostic tests for TTS, none has been shown to be sufficiently reliable for clinical use. The exacerbation of symptoms of TTS by standing and walking in many cases and the presence of agonizing symptoms despite having negative EDXTs have stimulated us to perform the EDXTs while challenging the foot in a biomechanical position that either stresses the neurovascular bundle or loads the foot in standing position.

As provocation of symptoms is usually subjective from one patient to the other, we considered evaluating the changes objectively using the three novel provocative EDXTs as the main judging parameters for the specificity and sensitivity of those tests.

Changes in ankle position were found to increase the pressure in the medial and lateral plantar tunnels even more than the pressure in the tarsal tunnel itself which can help in detecting more distal compression [10]. The positions of the ankle and subtalar joints were also the target of previous investigation by Abouelela and Zohiery who suggested the TCST for the diagnosis of TTS [6]. According to Trepman et al., the tarsal tunnel pressure increased by eversion or inversion of the ankle with a mean level of elevation of 32 mmHg and 17 mmHg respectively [11]. Also, Barker et al. concluded that pressure in the tarsal tunnel as well as medial and lateral plantar tunnels increased on doing dorsiflexion and eversion simulating pronation [10].

We agree with these studies that the provocation of symptoms in TTS patients could be due to changes in tension on the tibial nerve and surrounding structures during foot and ankle positions especially in pronation. External pressure reduces flow in the vessels supplying a nerve causing local ischemia and affecting the nerve axon ability to transmit action potentials [12–14]. In a clinical and operative study done by Mitsuo et al., local tenderness was exaggerated by dorsiflexion and eversion of the foot in 42 of 43 feet with symptoms suggestive of TTS, and it was induced in one foot in which it had been previously absent [15]. A Tinel’s test became more exaggerated in 41 feet, and the sign was induced in three feet in which it had been absent previously supporting our results of the validity of the EDCT in the diagnosis of TTS and explaining why we have had a considerable number of pronated feet among our TTS-positive cases.

The idea of the other two novel tests was to apply biomechanical loading force by acquiring standing position during electrophysiological testing which to the best of our knowledge is done for the first time.

The aggravation of patients’ symptoms during standing and walking in cases of TTS could be explained by the fact that the foot is the most dynamic organ of the body. During the stance phase, the subtalar joint undergoes eversion in the early phase and vice versa. Loss of joint motion, muscle weakness, sensory imperceptions, abnormal soft tissues, and bony misalignment could affect foot biomechanics during the gait cycle [16].

Since there is an agreement that the reduced amplitude and increased latency of the motor response are the most sensitive indicators of TTS, these were the main targeted variables during routine and novel testing as other EDX parameters as sensory nerve action potentials are hard to elicit (even in normal subjects) and frequently require averaging and conduction velocity is rarely affected except in late severe cases [6, 17].

The specificity of the routine diagnostic test was more for the LP than the MP if it is considered individually with the latency more specific for diagnosis than the amplitude this is in accordance with O’Brien and Byrden [2]. We recommend that NCSs for the diagnosis of TTS should include latency and amplitude of both MP and LP nerve motor response.

According to our results, the sensitivity of the routine EDXTs for MP and LP nerves in the diagnosis of clinically suspected TTS was only 60.9%. Routine EDXTs were able to detect TTS in only 28 patients, while they were negative in the remaining 18 patients, supporting our hypophysis that new tests are needed to detect those missed symptomatic cases. Our results were nearly in accordance with the results of Abouelela and El Zohiery who reported that the basic routine tests were positive in only 76.9% [6]. Also, the sensitivity for TTS diagnosis increased by the combination of more than one provocative test. The sensitivity increased on using LP or MP NCSs to be 78.3% and 82.6% respectively. The diagnostic sensitivity was further increased to reach a 100% on doing both LP and MP NCSs using the three new provocative tests, where affection of either amplitude or latency (increased distal latency and/or decrease in amplitude) in one of these new tests can be considered positive. The full diagnostic sensitivity reached 100% for the first time by using the battery of the three novel EDXTs in comparison to 85% by the clinical TCST by Abouelela and El Zohiery [6].

Tinel’s test was positive in 37 limbs while 9 limbs had negative Tinel’s test of whom 8 patients had negative routine NCSs while proved positive by the battery of novel tests. This gives the novel tests the advantage of diagnosing TTS in the absence of provocative clinical Tinel’s test.

Regarding the bilaterality of TTS, symptoms were found in 53% (16 patients with 32 feet) of our cases; this is in accordance with the results of Sodani et al. [18], who reported that bilateral symptoms in significant numbers of their patients 70%, but in contrast to Oh et al. and Urgan et al., who did not find bilateral symptoms among their study groups [19, 20]. This discrepancy might be attributed to the cause of TTS, where symptoms are more common to be bilateral in patients with idiopathic TTS and unilateral in patients with secondary TTS.

Methods to increase the sensitivity and hence decrease the false-negative test results such as the novel provocative tests (EDCT, DST, and SST) would be ideal to discover early affection. This would probably address treating TTS with conservative approach before the condition becomes chronic with irreversible sequels that makes surgical management inevitable. Future studies using the proposed provocative tests are needed to study the impact of flat foot deformities (congenital and acquired) and their relation to TTS.

### Study limitation

One of the limitations is conducting the study during the era of COVID-19 which affected patients flow rate. Also, many patients were excluded due to high BMI which might affect NCSs and patient stability during standing. Lastly, regarding the results of the routine and novel tests blinding was not done intentionally as the results are objective, moreover we were meticulous in performing the novel tests immediately after routine tests at the same sites marked on patients’ skin.

## Conclusion

There is an unmet need to consider new diagnostic tests for the diagnosis of TTS as according to our results the routine EDXTs has only 60% sensitivity. No single EDXT could be considered diagnostic; however, a battery of tests should be done. These provocative tests are sensitive, simple to apply, can imitate the biomechanical challenge that occurs during standing and walking. Using these novels tests has increased the diagnostic sensitivity of TTS reaching 100% thus, helping in the diagnosis of subclinical cases.

## Data Availability

All data generalized and/or analyzed during the current study are available from the authors upon reasonable request.

## Abbreviations

ADQ: Abductor digiti quinti
AH: Abductor hallucis
BMI: Body mass index
DS: Double stance
DST: Double stance test
EDX: Electrodiagnostic
EDXT: Electrodiagnostic test
EDCT: Eversion dorsiflexion compression test
EDB: Extensor digitorum brevis
LP: Lateral plantar
MP: Medial plantar
MNCSs: Motor nerve conduction studies
NCSs: Nerve conduction studies
REC: Research Ethics Committee
SS: Single stance
SST: Single stance test
TTS: Tarsal tunnel syndrome
TCST: Triple compression stress test
